# Challenges in Treatment Node Classification for Network Meta‐Analysis of Non‐Pharmacological Interventions: Insights From a Researcher Survey

**DOI:** 10.1111/jebm.70171

**Published:** 2026-07-30

**Authors:** Kansak Boonpattharatthiti, Deborah M. Caldwell, Nathorn Chaiyakunapruk, Teerapon Dhippayom

**Affiliations:** ^1^ The Research Unit of Evidence Synthesis Faculty of Pharmaceutical Sciences Naresuan University Phitsanulok Thailand; ^2^ Advancing Collaboration Translation, and Innovation for Value in Evidence Synthesis Hub of Talent Faculty of Pharmaceutical Sciences Naresuan University Phitsanulok Thailand; ^3^ Faculty of Pharmaceutical Sciences Burapha University Chonburi Thailand; ^4^ Population Health Sciences Bristol Medical School University of Bristol Bristol UK; ^5^ Department of Pharmacotherapy College of Pharmacy University of Utah Utah USA; ^6^ IDEAS Center Veterans Affairs Salt Lake City Healthcare System Utah USA; ^7^ Department of Social and Administrative Pharmacy Faculty of Pharmaceutical Sciences Chulalongkorn University Bangkok Thailand

**Keywords:** network meta‐analysis, node making, non‐pharmacological interventions, survey, treatment node

## Abstract

**Objective:**

Non‐pharmacological interventions (NPIs) are diverse and complex, making treatment node classification in network meta‐analyses (NMAs) particularly challenging. Accurate node classification is essential, as it directly affects the validity and applicability of NMA findings. The aim of this study was to explore current practices in treatment node classification in NMAs of NPIs and identify key challenges.

**Methods:**

An online survey was conducted among the first and corresponding authors of NMAs of NPIs published in first‐quartile journals over the past five years. Eligible authors were identified through a systematic search of PubMed, Embase, and CINAHL on February 4, 2025. A self‐administered questionnaire assessed experiences, perceptions, and recommendations regarding node classification. Quantitative data were summarized descriptively, and qualitative responses were analyzed thematically.

**Results:**

Of 471 eligible authors contacted, 32 responded (response rate: 6.8%). Most (43.8%) had 5–10 years of experience in evidence synthesis, and 81.3% had published 1–5 NMAs of NPIs. Nearly all reported difficulties in node classification due to within‐intervention and comparator variation, incorporating delivery features, balancing lumping versus splitting, and meeting the transitivity assumption. Respondents classified nodes by following prespecified protocols, relying on the characteristics of included studies, or using mixed approaches. While views on standard guidance were mixed, several respondents supported the development of simplified guidance for major research areas to support consistent node classification.

**Conclusions:**

Researchers encounter considerable difficulties in classifying treatment nodes in NMAs of NPIs. Findings highlight the need for prespecified and structured frameworks for certain types of NPIs to improve reproducibility, and reliability in future NMAs.

## 1 Introduction

Non‐pharmacological interventions (NPIs) encompass a wide range of approaches that focus on lifestyle changes, behavioral therapies, and physical techniques rather than medications. Like pharmacological interventions, NPIs aim to prevent, treat, or cure health conditions [1]. NPIs are recommended in clinical practice for various conditions, with primary studies providing evidence of their benefits in improving health and quality of life [2]. Their benefits are further reinforced through evidence syntheses, such as meta‐analyses and network meta‐analyses (NMAs).

NMA is a statistical technique used to compare multiple treatments in a single analysis and to provide the ranking and hierarchy of interventions [3]. A key consideration in NMA is the classification of comparative treatment options, often referred to as the node‐making process [4]. The term “node” represents the interventions being compared in NMA [3]. Typically, treatment nodes are predefined as part of the patient, intervention, comparison, and outcome (PICO) framework to prevent post‐hoc decisions about merging (lumping) or separating (splitting) treatments from influencing the results [5, 6]. Different approaches to the categorization of treatment nodes can influence the comparability of interventions and the validity of results [6], as shown in a study by James et al., which found that the way treatment nodes are classified impacts the accuracy and reliability of NMAs, with inadequate reporting potentially introducing bias and uncertainty in decision‐making [4].

The diversity of NPIs, in terms of components, delivery methods, intensity, and contextual factors, poses a significant challenge for classifying treatments into distinct nodes for NMA. Given the potential impact of node classification on the validity and reliability of NMA findings, along with existing concepts on complex interventions and the newly introduced “the theme, intensity, and provider/platform” (TIP) framework for node classification in NMA of NPIs [7, 8], it is essential to examine current practices. This will help identify any gaps, needs, or challenges that existing concepts and frameworks can address or determine whether a new node classification framework is needed. This study aims to examine how evidence synthesis researchers define and apply treatment node classification in NMAs of NPIs.

## 2 Methods

### 2.1 Study Design and Population

An online questionnaire survey was conducted among evidence synthesis researchers who had published NMAs of NPIs in high‐impact journals. Eligible participants were the first and corresponding authors of NMAs of NPIs published in first‐quartile (Q1) journals within the past five years, as classified by the SCImago Journal & Country Rank (2023) [9] in the fields of medicine, psychology, health professions, multidisciplinary sciences, and nursing.

We conducted a comprehensive search of PubMed, Embase, and Cumulative Index to Nursing and Allied Health Literature (CINAHL), on February 4, 2025, using the following keywords: “network meta‐analysis,” “mixed‐treatment comparisons,” and “indirect comparisons,” limiting the publication date to the past five years to identify eligible NMA studies.

### 2.2 Recruitment

We invited the first and corresponding authors of the identified NMAs of NPIs to participate in this survey. If an author had published more than one eligible article during this period, only one invitation was sent to avoid duplication. Survey invitations were distributed via email and included the purpose of the study, the estimated time to complete the questionnaire, and a link to the self‐administered survey hosted on Google Forms. Additional recruitment efforts were made through professional networks, including outreach to members of the Society for Research Synthesis Methodology, to enhance coverage and diversity. A follow‐up reminder email was sent approximately ten days after the initial invitation to encourage participation.

### 2.3 Instrument and Measures

A self‐administered questionnaire was developed to explore researchers’ experiences, perceptions, and recommendations regarding treatment node classification in NMAs of NPIs. It comprised three parts covering respondents’ research background and experience with node classification, their awareness and perceptions including views on future guidance, and demographic information. The questionnaire included both closed‐ and open‐ended items addressing criteria for defining treatment nodes, challenges encountered, strategies for merging or splitting nodes, awareness of existing guidance, views on standardization, and suggestions for improving reporting transparency.

The questionnaire was collaboratively drafted and refined by the research team. To assess its clarity and feasibility, a pilot test was conducted with researchers experienced in NMAs of NPIs. Participants completed the survey and provided feedback on question clarity, response options, and overall survey structure. Revisions were made based on this feedback. The details of the questionnaire are provided in Text S1.

### 2.4 Data Analysis

Responses to closed‐ended questions were summarized using descriptive statistics. Categorical variables were reported as frequencies and percentages. Data were analyzed using Microsoft Excel. Responses to open‐ended questions were examined using a thematic analysis approach. K.B. and T.D. independently reviewed and coded the responses to identify emerging themes. Discrepancies in coding and theme identification were resolved through discussion and consensus with reference to the original responses. The thematic analysis focused on key issues related to treatment node classification and researchers’ recommendations for improvement.

### 2.5 Ethical Consideration

This study was conducted in accordance with the Declaration of Helsinki. This study was approved by the institutional review board of the University of Utah (IRB_00189074). Participation in the survey was voluntary, with informed consent implied through survey completion; no personal identifiers were obtained, and responses were anonymized to ensure confidentiality.

## 3 Results

### 3.1 Response Rate

We identified 5349 NMAs after matching the journal titles to the Q1 list. We screened these records against the eligibility criteria, which required that the article be an NMA of NPIs, without restrictions on the types of studies included in the NMA. A total of 677 NMAs of NPIs were identified. After removing duplicate contacts, we obtained contact information for the first and/or corresponding authors of 629 articles. Email invitations were sent to these authors; however, 158 emails were either invalid or unreachable, resulting in 471 authors successfully receiving the invitation. Of these, 32 authors responded, yielding a response rate of 6.8%.

### 3.2 General Characteristics of the Respondents

Out of 32 respondents, 18 were male (56.3%). The respondents’ ages ranged from under 30 to over 60 years, with the majority in the 30–60 age group (*n* = 23; 71.9%). Most respondents (*n* = 27; 84.4%) reported working in the fields of systematic review and meta‐analysis, followed by epidemiology (*n* = 9; 28.1%) and biostatistics (*n* = 7; 21.9%). Ten respondents (31.3%) had more than 10 years of experience in evidence synthesis, 14 (43.8%) had 5–10 years of experience. Twenty‐six respondents (81.3%) had published 1–5 NMAs of NPIs, while three (9.4%) had published more than 10 NMAs (Table 1).

**TABLE 1:** General characteristics of respondents.

General characteristics of respondents	Total (*n* = 32, %)
Age	
<30 years	5 (15.6)
30–60 years	23 (71.9)
>60 years	4 (12.5)
Field of expertise	
Biostatistics	7 (21.9)
Epidemiology	9 (28.1)
SR/MA and data sciences	28 (87.5)
Clinical backgrounds^*^	25 (78.1)
Years of experience in evidence synthesis	
1–5 years	8 (25.0)
5–10 years	14 (43.8)
>10 years	10 (31.3)
Number of NMAs of NPIs	
1–5 papers	26 (81.3)
5–10 papers	3 (9.4)
>10 papers	3 (9.4)

Abbreviations: MA, meta‐analysis; NMA, network meta‐analysis; NPI, non‐pharmacological intervention; SR, systematic review.

* Alliance health professionals, clinical and public health medicine, behavioral and mental health sciences.

### 3.3 Research and Experience in Treatment Node Classification

#### 3.3.1 Difficulties in Classifying NPIs Into Treatment Nodes for NMA

All respondents had experience classifying treatment nodes in an NMA of NPIs. Thirty‐one respondents (96.9%) reported difficulties with this task. Twenty‐one respondents (65.6%) considered treatment node classification moderately difficult, while nine respondents (28.1%) found it very difficult (Table 2). To further understand these challenges, participants were also invited to elaborate in an open‐ended question about their difficulties with treatment node classification. The difficulties researchers encountered included variations within interventions and controls, deciding whether to lump or split interventions, and meeting the transitivity assumption.

**TABLE 2:** Background and perception on treatment node classification in network meta‐analyses of non‐pharmacological interventions (*n* = 32).

Question	All (*n* = 32, %)
Encountered difficulties in classifying NPIs into treatment nodes for NMA	
Yes	31 (96.9)
Level of difficulty in classifying NPIs into treatment nodes for NMA	
Median [IQR]	3 [3, 4]
Not difficult at all	0 (0)
Slightly difficult	2 (6.3)
Moderately difficult	21 (65.6)
Very difficult	9 (28.1)
Extremely difficult	0 (0.0)
Criteria used to define treatment nodes in NMAs of NPIs	
Type of intervention	31 (96.9)
Mode of delivery (e.g., frequency, provider, and platform)	19 (59.4)
Intensity or dosage	13 (40.6)
Others	5 (15.6)
Typical approach used to define treatment nodes before conducting the NMA	
Pre‐specified before reviewing the data	11 (34.4)
Decided after reviewing the available data	3 (9.4)
Combination of both	18 (56.3)
Others	0 (0.0)
Importance of standardized node classification (transparency, pre‐specification, reporting guidelines, or a structured framework) for improving the reliability of NMAs	
Median [IQR]	4 [4, 5]
Strongly disagree	0 (0.0)
Disagree	0 (0.0)
Neither agree nor disagree	5 (15.6)
Agree	14 (43.8)
Strongly agree	13 (40.6)
Suggested standardization approaches to improve the reliability of node‐making in NMAs of NPIs	
Pre‐specification of the node‐making process	20 (62.5)
Reporting guidelines for node classification	23 (71.9)
Framework to structure the node‐making process	22 (68.8)
Others	4 (12.5)
Awareness of guidelines, frameworks, or recommendations for treatment node classification in NMA	
Yes	10 (31.3)
Interest in using a framework that incorporates delivery characteristics for NPI classification	
Yes	29 (90.6)
Agreement that guidance on using the framework would be beneficial	
Median [IQR]	4 [4, 5]
Strongly disagree	0 (0.0)
Disagree	0 (0.0)
Neither Agree nor Disagree	2 (6.3)
Agree	17 (53.1)
Strongly agree	13 (40.6)

Abbreviations: IQR, interquartile range; NPI, non‐pharmacological intervention; NMA, network meta‐analysis.

##### 3.3.1.1 Theme 1: Variations Within Interventions and Control

Respondents also reported concerns regarding variability across interventions and comparators. This variability complicated the process of defining treatment nodes.

One frequently mentioned issue was the lack of standard definitions for interventions. Several participants stressed that interventions are inconsistently labeled across studies, making classification difficult. As one participant explained, “There are no standard names of the interventions; names are used inconsistently across different studies” (Respondent 4). Another respondent similarly pointed out that “NPIs require researchers to define the classification criteria themselves” (Respondent 17), highlighting the absence of established terminology or guidance.

In addition, many respondents described problems with the insufficient description of interventions and comparators in trial reports. Some intervention descriptions lacked essential detail, limiting the ability to accurately assign them to a node. As one respondent stated, “Description of the interventions is not always detailed” (Respondent 4). While another added: “Descriptions of treatments are sometimes short and descriptions of comparators are oftentimes lacking (labeling the comparator simply treatment as usual, usual care, attention control, or control)” (Respondent 31).

The complexity of NPIs also emerged as a key concern. Many NPIs are multi‐component interventions, which make it difficult to decide whether to group them under a single node or treat them as separate entities. One participant noted: “Many NPIs are complex interventions with multiple components. This complexity makes it challenging to determine whether interventions should be grouped together or considered as distinct nodes” (Respondent 8). Others pointed to the difficulty of classifying combinations of interventions, such as deciding how to handle “choices regarding nodes which are a composite of several interventions” (Respondent 13).

##### 3.3.1.2 Theme 2: Deciding Whether to Lump or Split Interventions

Respondents described the challenge of deciding whether to combine similar interventions into a single node (lumping) or separate them into distinct nodes (splitting), particularly when interventions differ in delivery characteristics. Variations in intensity, duration, and mode of delivery were frequently cited as complicating factors. One respondent highlighted the complexity of grouping interventions: “The decision to group with the intervention type, dose, and interventionist” (Respondent 20). Another explained, “In an NMA aiming to identify the most effective obesity prevention program, classifying physical activity interventions becomes complex due to such variations in delivery, which must be carefully considered in node classification” (Respondent 17).

Concerns were also raised about the inconsistent or overly broad classifications could weaken both the clinical relevance and methodological rigor of NMAs. As one participant cautioned, “Inconsistent or overly broad classifications may compromise the clinical relevance and validity of the analysis” (Respondent 8).

Many respondents underscored the importance of expert input to ensure that treatment nodes captured meaningful clinical distinctions. However, they also recognized the potential drawbacks of over‐relying on content experts without sufficient methodological understanding. One respondent stated, “expert input is often essential to ensure that treatment nodes are defined in a way that reflects meaningful clinical distinctions” (Respondent 8), while another noted the need for balance: “Need to compromise between too many nodes and too heterogeneous treatments, relied on content experts with insufficient insight into methods” (Respondent 18).

##### 3.3.1.3 Theme 3: Meeting the Transitivity Assumption

Some respondents emphasized the need to define treatment nodes in a way that supports the transitivity assumption, a key requirement for valid indirect comparisons in NMAs. They noted that variations in intervention characteristics can threaten this assumption. One respondent explained: “Ensuring that all nodes meet the transitivity assumption (or rather whether nodes/comparison are more likely to be assessed in specific subsets of patients)” (Respondent 7). These concerns highlight the importance of aligning node definitions with underlying effect modifiers to maintain methodological integrity.

#### 3.3.2 Strategies for Classifying Treatment Nodes in NMAs of NPIs

Thirty‐one respondents (96.9%) classified the NPIs based on the type of intervention, while 19 (59.4%) and 13 (40.6%) classified them based on the mode of delivery and intensity, respectively (Table 2). In addition to the criteria used, respondents also differed in when they applied these classifications during the review process. Eleven respondents (34.4%) classified treatment nodes using pre‐specified criteria before reviewing the data, while three respondents (9.4%) did so after reviewing the available data. Eighteen respondents (56.3%) used both approaches for treatment node classification.

Respondents reported a variety of strategies for deciding how to merge or split treatment nodes in NMAs of NPIs. Three broad themes emerged: following a pre‐specified protocol, decisions based on included papers, and mixed approaches.

##### 3.3.2.1 Theme 1: Following a Pre‐Specified Protocol

Several respondents emphasized that their decisions were primarily guided by the study hypothesis and research questions. As one participant stated, “The most important is your study hypothesis” (Respondent 1), while another emphasized, “According to the protocol. And the protocol should follow the research question” (Respondent 3). In addition to study aims, some respondents highlighted the importance of theoretical considerations in shaping node definitions. For example, one respondent explained that their approach was “based on theoretical considerations (e.g., assumed mechanism of action, label of treatments according to the authors description)” (Respondent 31).

Many respondents reported that their decisions relied on evidence from prior studies. They described approaches such as “Based on prior systematic reviews, particularly where splitting treatment nodes is likely to be a way to resolve heterogeneity observed in previous evidence of NPIs” (Respondent 7) and “Normally, based on pre‐defined modalities that have been studied in previous research” (Respondent 9).

##### 3.3.2.2 Theme 2: Decisions Based on Included Papers

Many respondents reported that the availability and level of detail in the included studies significantly influenced how treatment nodes were defined. Interventions were often grouped based on shared features such as content, techniques, or mode of delivery. As one respondent noted, “Based on the similarity of the interventions” (Respondent 4), while another added, “Based on the contents/techniques of the intervention” (Respondent 6). In some cases, researchers employed specific strategies to enhance consistency across nodes, including independent assessments to increase homogeneity. One respondent described this process as: “Have two people independently try to increase homogeneity within a node” (Respondent 14).

Clinical reasoning also played an important role in guiding decisions about whether to lump or split interventions. Some respondents relied on their professional judgment to determine whether it was appropriate to group treatments together, particularly when making distinctions that were clinically meaningful. One respondent noted that their decisions were based “on clinical impact” (Respondent 8), while another emphasized the importance of assessing “whether it makes clinical sense to group treatments into a node” (Respondent 10). These responses reflect how real‐world clinical relevance often shaped node classification. In addition to individual judgment, many respondents involved external experts to support or validate their decisions. This included formal consultation processes or informal expert feedback. As one participant explained, “We engaged a group of experts to help classify treatment types” (Respondent 11), while another stated their approach was “based on expert feedback” (Respondent 13).

##### 3.3.2.3 Theme 3: Mixed Approaches

Respondent reported combining prespecified classification with adjustments made during the review process if required. As one described, “However, if the a priori classification creates problems (e.g., networks become unconnected, lack of closed loops), we reconsider classification, which does not necessarily mean that we adapt/change the classification, but we check if an alternative is possible which might overcome particular problems/challenges with regards to the analysis based on the initially planned classification. Sometimes classification is adapted in this process. In most cases we describe/report the different analyses at least in the supplement” (Respondent 31).

### 3.4 Awareness, Perception on Standardization of Treatment Node Classification

Twenty‐seven respondents (84.4%) agreed or strongly agreed that standardized node classification, which covers transparency, pre‐specification, reporting guidelines, or a structured framework is important for improving the reliability of NMAs. Most respondents supported specific strategies: 23 (71.9%) favored reporting guidelines, 22 (68.8%) a structured framework, and 20 (62.5%) pre‐specification. However, 22 (68.8%) were unaware of any existing guidelines, frameworks, or recommendations. A large majority (29; 90.6%) expressed interest in a framework that incorporates delivery characteristics of NPIs, with 17 (53.1%) agreeing, 13 (40.6%) strongly agreeing, and 2 (6.3%) neutral about the need for accompanying guidance (Table 2).

### 3.5 Guidance on Treatment Node Classification

Respondents provided a range of suggestions for improving treatment node classification, which were grouped into three themes.

#### 3.5.1 Theme 1: Need for Guidance in Classification (Mixed Perspectives)

There were mixed views on whether standardized guidance for node classification is feasible or desirable. Some respondents expressed doubts about the applicability of a universal approach, noting that different study questions may require different classification strategies. As one respondent stated, “I am not sure there could be a standardized approach across different study questions” (Respondent 18). While some respondents questioned the feasibility of applying a single standardized approach across diverse study contexts, others saw value in developing simplified, field‐specific guidance to support more consistent node classification. These respondents recognized that although full standardization may not be practical, having basic recommendations tailored to major research areas could still help streamline decisions. As one participant suggested, “It would be very beneficial for future research to consolidate some of the guidelines for major research areas into a simple node differentiation guideline” (Respondent 32).

In addition, several respondents noted that transparency in how treatment nodes are classified is more important than strictly following fixed or universal rules. They emphasized the importance of clearly explaining the rationale behind classification decisions, especially in the absence of standardized guidance. As one respondent stated, “Not necessarily; crucial is that it is reported transparently” (Respondent 24). This reflects a shared view that, even without uniform guidelines, transparent reporting can enhance the credibility and reproducibility of NMAs.

There were calls for a more structured approach to guide decision‐making. This included step‐by‐step procedures outlining how to prioritize approaches, handle heterogeneity or inconsistency, and report decisions clearly. As one participant proposed, “Finally, I would like a stepped procedure, recommending which approach to prioritize, how to continue in case of heterogeneity/inconsistency, and including explicit recommendations for reporting” (Respondent 31).

#### 3.5.2 Theme 2: Ensure Clinical Relevance and Evidence Alignment

Respondents emphasized the need to balance pre‐specification with flexibility when defining treatment nodes. One participant recommended developing a structured plan during the study design phase, stating “I think it would be useful to create a predefined procedure for classifying treatment nodes during the study planning phase” (Respondent 17).

Some respondents noted that delivery characteristics, such as the intensity, frequency, and how an intervention is administered, should only be included in node definitions if they are clearly justified by the research hypothesis. As one explained, “If you need no classification for both the active complainant of the intervention and the form of delivery, you'd need to have some hypothesis for why in most cases the interaction between these matters” (Respondent 14).

Several suggestions emphasized the need for aligning classification systems with established clinical standards and frameworks. One respondent stated, “It is difficult to rely solely on the classification of primary study authors. Therefore, authors of NMAs need their own classification system. However, this classification system must be consistent with other clinical guidelines or frameworks, e.g. physical therapy interventions should be in accordance with the World Confederation for Physical Therapy definition” (Respondent 30).

In addition, some respondents recommended involving practitioners or clinical experts in the classification process. They believed that expert feedback could help ensure that nodes reflect real‐world understanding of intervention similarity. One respondent suggested, “My first suggestion would be, to include considerations about the comparator as well, as this aspect is overlooked in NMAs (and MAs) in many cases, in my view. Feedback from practitioners or experts from (clinical) practice could be helpful as well (for instance, if they considered two interventions too similar to be considered individually, one could combine them)” (Respondent 31).

#### 3.5.3 Theme 3: Technical and Methodological Considerations

Some respondents highlighted the value of independent decision‐making, with one noting, “Pre‐specification is the key. It is also desirable that pairs of researchers decide the node, as in the screening process” (Respondent 3). Others recommended considering the structure of the evidence network, as one explained, “Perhaps it would also be important to consider the number of connections between the different nodes trying to avoid isolated nodes” (Respondent 9).

## 4 Discussion

Node‐making is influenced by multiple factors, including the research aim, available evidence, and feasibility of analysis. Our findings indicate that researchers have used several approaches to define or classify treatment nodes in NMAs of NPIs, including using prespecified criteria, making decisions based on included studies, or applying mixed approaches. However, they encountered numerous difficulties and concerns, such as incorporating delivery characteristics into node definitions, addressing the complexity and multiple components of NPIs, and deciding whether to lump or split treatment nodes. NPIs are often heterogeneous, multi‐component, and poorly described in primary studies, making it difficult for researchers to classify them into appropriate treatment nodes in NMAs. These findings provide insight into the current practices researchers use to define treatment nodes and highlight the common difficulties they encounter, underscoring the need for solutions or guidance to improve the reliability of treatment node classification in NMAs of NPIs.

Researchers reported difficulties in deciding whether to use a lumping or splitting approach when classifying treatment nodes. Generally, the lumping approach risks masking meaningful distinctions, while the splitting approach may fragment networks and reduce statistical power. Davies et al. found that NMAs of public health interventions tended to lump interventions into broader categories, although some studies used splitting based on intervention components or delivery features [10]. Furthermore, Del Giovane et al. found that NMAs combining both pharmacological and non‐pharmacological interventions tend to lump the differences contexts of control groups into the same treatment node [11]. There is no universally accepted rule for when to lump or split interventions. However, this decision can significantly influence the results. Xing and Lin empirically showed that varying treatment classifications, whether grouping similar interventions together or analyzing them separately, produced considerable changes in effect estimates and model performance across multiple NMAs [6].

Incorporating delivery characteristics of NPIs (such as intensity, duration, and provider) into treatment nodes were also identified as a key challenge in treatment node classification. These delivery features are conceptually analogous to dose and route of administration in pharmacological interventions [8], where different doses or routes of the same drug can yield distinct treatment effects. Del Giovane et al. demonstrated that decisions on whether to classify treatments by dose in NMA substantially affect heterogeneity, consistency, and ultimately the estimated effects [12]. These findings highlight that how delivery characteristics are classified in NPIs can meaningfully influence treatment node definitions and, in turn, the outcomes of the analysis.

Despite these difficulties, researchers have attempted to address them using multiple approaches. Most respondents emphasized the importance of aligning node classification with the study hypothesis and a pre‐specified protocol, while others suggested drawing on previous systematic reviews, and expert consultation. Additionally, some researchers suggested a balance between pre‐specification and the flexibility needed to accommodate study characteristics and the available network structure. These attempts might cause an overlapping of published NMAs on the same research question [13].

Several frameworks, such as intervention component analysis (ICA) [14] and the context and implementation of complex interventions framework (CICI) [15] provide valuable insights into implementing complex interventions by highlighting key features, context, and processes. However, they are difficult to apply directly to node classification in NMAs, as they were not designed for this purpose. Alternatively, the Theme, Intensity, and Provider/platform (TIP) framework was developed specifically to support node classification in NMAs by incorporating the delivery characteristics of NPIs into treatment nodes. Nearly all respondents expressed interest in frameworks that incorporate delivery features, highlighting the potential value of structured approaches like the TIP framework for enhancing transparency, reproducibility, and clinical interpretability. Nonetheless, they emphasized that guidance on treatment node classification should remain flexible and sensitive to context.

Classifying treatment nodes in NMAs of NPIs remains a major methodological challenge. Researchers should clearly report the rationale for node definitions, explain how delivery features and comparators are considered, and justify decisions to lump or split interventions. In the absence of standardized guidance, node classification should involve relevant stakeholders and balance pre‐specification with flexibility to reflect study characteristics, network structure, and clinical relevance. Embedding these transparent practices in protocols and reporting standards will enhance the reproducibility, credibility, and clinical value of NMAs of NPIs.

Our study has several limitations. First, the response rate was relatively low, which may limit the representativeness of our findings and raise the possibility of selection bias. Therefore, the results should be interpreted as reflecting the perspectives of those who chose to participate in the survey. However, those who did participate provided detailed and thoughtful responses, particularly to the open‐ended question, offering valuable insights into their practices, perceptions, and suggestions for the future. Second, because the survey was conducted by email, there were no opportunities to clarify or probe responses, and some participants had limited experience with NMAs of NPIs. Third, because the survey was conducted anonymously, we did not collect identifying information linking responses to specific NMAs. Consequently, we were unable to compare NMA‐level characteristics between respondents and non‐respondents. This limits our ability to formally assess potential non‐response bias or examine whether respondents differed systematically from non‐respondents in terms of study characteristics, such as intervention type or methodological features. As a result, the generalizability and external validity of our findings may be limited. Fourth, because the survey was administered via Google Forms, participation from authors located in China may have been limited due to restricted access to Google services. This may have resulted in underrepresentation of authors from this region and further reduced the generalizability of our findings. Finally, although thematic analysis of open‐ended responses allowed us to identify common patterns, the nature of brief and context‐limited written comments limited the depth of interpretation compared with interviews or focus groups.

This study provides valuable insights into the practice and perception of a small sample of evidence synthesis researchers. The findings suggest that researchers used a variety of strategies to classify treatment nodes in NMAs of NPIs, including prespecified protocols, decisions based on the characteristics of included studies, and mixed approaches that combine predefined rules with adjustments during the review process. It also highlights persistent challenges, such as the complexity of NPIs, the lack of standardized criteria, and the ongoing “lumping versus splitting” dilemma. However, given the modest response rate, these findings should be interpreted with caution. While there is support for pre‐specification and structured frameworks to improve reliability, guidance and consensus‐driven frameworks should be tailored to specific types of NPIs, as a universal approach is unlikely to be suitable given their complexity and heterogeneity. Broader perspectives from a larger pool of NMA researchers are needed to validate these findings and to inform the development of practical guidance on node classification for NPIs.

## Funding

This study is supported by the National Research Council of Thailand (N41A670345).

## Conflicts of Interest

All authors declare no conflicts of interest.

## Supporting information




**Text S1**: Questionnaire.
