# Supplementary material for: Challenges in Treatment Node Classification for Network Meta‐Analysis of Non‐Pharmacological Interventions: Insights From a Researcher Survey
Source: J Evid Based Med. 2026 Jul 30;19(3):e70171. doi: 10.1111/jebm.70171 (PMC13613242; doi:10.1111/jebm.70171)
Supplement: Supplementary file 1 [file JEBM-19-0-s001.docx]

Supplementary materials

Text S1: Questionnaire

**Form Description:** We are gathering insights from evidence synthesis researchers on treatment node classification in network meta-analysis (NMA) of non-pharmacological interventions (NPI).

NPIs are science-based, non-invasive interventions designed to prevent, treat, or cure health issues through products, methods, programs, or services. Examples of NPIs include psychosocial interventions, music interventions, diet, exercise, and public health programs.

**Objective:** To examine how evidence synthesis researchers define and apply treatment node classification in NMAs of NPI.

**Instruction:** Please select the most appropriate answer.

*************

**Part 1: Research and Experience in Treatment Node Classification**

1. Have you ever been involved in classifying treatment nodes in an NMA of NPI?

☐ Yes	☐ No

2. If you answered “Yes” to Question 1, please indicate the number of NMAs of NPIs you have worked on:

☐ 1-5 papers	☐ 5-10 papers	☐ More than 10 papers

3. Have you ever encountered difficulties in classifying NPIs into treatment nodes for NMA?

☐ Yes	☐ No

4. Please describe any difficulties or concerns you have encountered regarding treatment node classification in NMAs of NPIs.

_____________________________________________________________________

5. How difficult is it for you to classify NPIs into treatment nodes for NMA?

| ☐ | ☐ | ☐ | ☐ | ☐ |
| --- | --- | --- | --- | --- |
| Not difficult at all | Slightly difficult | Moderately difficult | Very difficult | Extremely difficult |

6. What criteria do you use to define treatment nodes in NMAs of NPIs? (Multiple choice, select all that apply)

☐ Type of intervention

☐ Mode of delivery (e.g. frequency, provider and platform)

☐ Intensity or dosage

☐ Other (please specify)

7. Do you typically pre-specify the criteria for defining treatment nodes before conducting the NMA, or is the decision influenced by the data available during the review process?

☐ Pre-specified before reviewing the data

☐ Decided after reviewing the available data

☐ Combination of both

☐ Other (please specify):___________

8. How do you typically decide to merge or split treatment nodes in NMAs of NPIs?

________________________________________________________________________

**Part 2: Awareness, Perception, and Future Guidance on Node Classification**

9. Do you think standardized node classification — in terms of transparency of the node-making process, pre-specification of the node-making process, reporting guidelines for node classification, or a framework to structure the node-making process — is important for improving the reliability of NMAs?

| ☐ | ☐ | ☐ | ☐ | ☐ |
| --- | --- | --- | --- | --- |
| Strongly Disagree | Disagree | Neither agree nor disagree | Agree | Strongly Agree |

10. What kind of standardization approach do you think would improve the reliability of node-making process for NMAs of NPIs? (Multiple choice; select all that apply)

☐ Pre-specification of the node-making process

☐ Reporting guidelines for node classification

☐ Framework to structure the node-making process

☐ Other (please specify)

11. Are you aware of any guidelines, frameworks or recommendations for treatment node classification in NMA?

☐ Yes	☐ No

12. If there is a framework that assists in classifying NPIs by incorporating how the intervention is delivered, would you like be interested in using it?

☐ Yes	☐ No

13. Do you agree that guidance on using such a framework would be beneficial?

| ☐ | ☐ | ☐ | ☐ | ☐ |
| --- | --- | --- | --- | --- |
| Strongly Disagree | Disagree | Neither agree nor disagree | Agree | Strongly Agree |

14. Do you have any suggestions for improving treatment node classification in NMAs of NPIs?

**Part 3: Demographic Information**

15. Affiliation and country

_____________________________________________________________________

16. Gender

☐ Male

☐ Female

☐ Prefer not to answer

17. Age

☐ Under 30 years

☐ 30-39 years

☐ 40-49 years

☐ 50-59 years

☐ 60 years and over

☐ Prefer not to say

18. Field of expertise: (Multiple choice, select all that apply)

☐ Epidemiology

☐ Biostatistics

☐ Systematic Review & Meta-analysis

☐ Health Economics

☐ Other (please specify)

19. Years of experience in evidence synthesis:

☐ 1-5 years	☐ 5-10 years	☐ More than 10 years
